# Unraveling the Complex Nexus of Human Papillomavirus (HPV) in Extragenital Keratinocyte Skin Tumors: A Comprehensive Analysis of Bowen’s Disease and In Situ Squamous-Cell Carcinoma

**DOI:** 10.3390/jcm13041091

**Published:** 2024-02-15

**Authors:** Claudio Conforti, Chiara Retrosi, Marina Agozzino, Caterina Dianzani, Ermanno Nardon, Anselmo Oliveri, Eros Azzalini, Stefania Guida, Giovanni Pellacani, Giovanni Di Lella, Franco Rongioletti, Iris Zalaudek, Serena Bonin

**Affiliations:** 1 IDI-IRCCS, Dermatological Research Hospital, 00167 Rome, Italy; claudioconforti@yahoo.com (C.C.); chiara.retrosi@gmail.com (C.R.); g.dilella@idi.it (G.D.L.); 2 Dermatology Clinic, Maggiore Hospital, Piazza Ospitale 1, 34125 Trieste, Italy; marinaagozzino@gmail.com (M.A.);; 3 Department of Plastic, Reconstructive and Cosmetic Surgery, Dermatology Section, Campus Bio-Medico University Hospital, 00128 Rome, Italy; c.dianzani@policlinicocampus.it; 4 Department of Medical Sciences (DSM), University of Trieste, 34149 Trieste, Italyanselmo.oliveri@studenti.units.it (A.O.); eazzalini@units.it (E.A.); sbonin@units.it (S.B.); 5 Dermatology Clinic, IRCCS San Raffaele Hospital, Vita-Salute University, 20132 Milan, Italy; rongioletti.franco@hsr.it; 6 Dermatology Clinic, Department of Clinical Internal, Anesthesiological and Cardiovascular Sciences, Sapienza University of Rome, 00196 Rome, Italy; pellacani.giovanni@gmail.com

**Keywords:** human papillomavirus, Bowen’s disease, squamous-cell carcinoma, keratinocyte skin tumors

## Abstract

This comprehensive study delves into the intricate landscape surrounding the role of human papillomavirus (HPV) in extragenital keratinocyte skin tumors, specifically exploring Bowen’s disease (BD) and in situ squamous-cell carcinoma (iSCC). Through a multifaceted examination, this research study elucidates the nuanced interplay of HPV, gender dynamics, anatomical site variations, and potential implications for the etiopathogenesis of these malignancies.

## 1. Introduction

The role of human papillomavirus (HPV) in keratinocyte skin tumors has been studied in recent years, with controversies surrounding its etiopathogenetic and/or cofactor role in the occurrence of these malignancies. Actinic keratoses (AK) are mostly positive for beta and gamma HPV genera, and only a few are positive for alpha subtypes [1]. Similarly, in squamous-cell carcinomas (SCCs), especially the beta [2,3] and gamma genera [4,5] have been identified, in contrast to Bowen’s disease (BD), where a greater occurrence of the alpha and beta genera has been detected [6] and in contrast to keratoacanthoma (KA), where the alpha, beta, and gamma HPV genera are present to a similar extent [1]. In addition to gender differences in the various keratinocyte tumors, different frequencies of HPV have been shown: the highest frequency was found by several studies in keratoacanthoma (around 90%), suggesting a cofactor role of HPV in its occurrence and/or exponential growth [7]; in contrast, SCC and BD have significantly lower frequencies (around 50%) than KA, supporting the primary role of UV rays in their etiopathogenesis. However, a new clinical classification has proposed [8] to use the term BD for tumors arising on non-photodamaged skin while reserving the term iSCC for tumors localized on photo-exposed and photodamaged skin; from a histological and dermatoscopic point of view, there are no differences between BD and iSCC, and indeed the two are used as synonyms [9].

On dermoscopy, they present glomerular vessels on an erythematous background, and histologically, they are characterized by full-thickness keratinocyte atypia and architectural and cellular atypia with apoptotic cells. With the aim of assessing whether UV rays are the main cause of these neoplasms or whether there is a viral cause associated with HPV, in this work, we performed a distinction between lesions arising on photodamaged skin and on non-photoexposed skin referring to the two forms by the terms iSCC and BD, respectively. By using this terminology, therefore, a series of BD and iSCC cases were compared to assess whether there is a difference in HPV frequency and/or gender expression between the two malignancies.

### 1.1. Overview of Human Papillomavirus (HPV)

Human papillomavirus (HPV) is a group of more than 200 DNA viruses that can infect the skin and mucosa. Among them, there are some forms defined as high-risk because a persistent infection is the main risk factor for the development of specific neoplasms, such as cervical cancer [9]. In detail, high-risk HPV genome integration is associated with persistent infection, which can lead to cancer progression [10].

HPV is a non-enveloped circular double-stranded DNA virus that belongs to the Papillomaviridae family. The virus has a selective tropism for keratinocytes located on the skin and mucous membranes. Lesions in the epithelium are the route for HPV to infect epithelial cells, where it enters and spreads to the basal layer [11]. Most HPV infections remain clinically undetectable, while in other cases, the presence of HPV can result in the formation of skin lesions such as warts, precancerous lesions, and tumors.

The carcinogenic risk being posed by HPV depends on the integration of the virus and the virus E6 and E7 oncoproteins. E6 and E7 gene products have been shown to be primarily responsible for the cellular transformation process. E6 and E7 encode for oncoproteins that alter cellular regulatory mechanisms, promoting cell proliferation and division, along with the activation of host defense evasion mechanisms [12]. The E6 gene product of high-risk HPV types, such as HPV-16 and HPV-18, inhibits p53, a tumor suppressor protein [13,14], and BAK. Both of these are key regulators of apoptosis. Through the formation of a complex with E6, p53 is targeted for degradation, leading to the loss of its tumor-suppressive functions. The inactivation of p53 by E6 allows infected cells to bypass cell cycle checkpoints and evade apoptosis, facilitating the persistence of infected cells and contributing to the oncogenic potential of high-risk HPV types. By positively affecting telomerase and the SRC kinase family, E6 promotes cell proliferation and, thus, cellular immortalization [10].

Similarly, the E7 gene encodes the E7 oncoprotein, which inhibits the retinoblastoma (Rb) tumor suppressor protein, leading to the release of E2F transcription factor that drives the expression of genes required for cell cycle progression [15,16]. This also determines p16 upregulation, a cyclin-dependent kinase inhibitor inactivation that acts as a tumor suppressor. The overall consequent dysregulation of the cell cycle leads to uncontrolled cellular proliferation, a hallmark of cancer. Additionally, E7 also stimulates cyclin A and E production, inactivates the cyclin-dependent kinase inhibitors p21 and p27, and promotes cell proliferation [10].

The integration of HPV DNA into the host genome is a crucial step in the progression of HPV-associated cancers. Unlike episomal replication, where the viral genome exists as an independent unit within the host cell nucleus, integration involves the physical insertion of HPV DNA into the host’s chromosomal DNA. This integration is more frequently observed in high-risk HPV infections and is associated with a higher risk of malignant transformation [17,18,19].

The molecular mechanisms underlying HPV DNA integration are complex and not fully understood. The viral E2 protein, which normally acts as a transcriptional repressor and regulator of viral replication, is disrupted during integration. The loss of E2 function results in increased expression of E6 and E7 oncoproteins, contributing to the cellular transformation process. Integration events often disrupt the E2 open reading frame, leading to uncontrolled expression of E6 and E7 [17,20].

Additionally, the integration of HPV DNA into the host genome can result in the disruption of host genes and chromosomal instability. The random nature of integration events makes it challenging to predict the specific genes affected, but alterations in key regulatory pathways can contribute to the malignant potential of infected cells.

Understanding the virology of HPV, particularly the roles of E6, E7, and DNA integration, has significant clinical implications. The identification of these viral factors as key players in oncogenesis has paved the way for the development of therapeutic strategies targeting specific aspects of the HPV life cycle.

One approach involves the development of vaccines that target the major high-risk HPV types, such as HPV-16 and HPV-18, responsible for most cervical cancer cases. Vaccination programs have proven effective in preventing HPV infection and reducing the incidence of associated cancers.

Furthermore, the development of antiviral drugs targeting specific stages of the HPV life cycle, including viral entry, replication, and gene expression, is an active area of research. Small-molecule inhibitors that selectively target viral oncoproteins such as E6 and E7 are being explored as potential therapeutic agents to disrupt the oncogenic activities of these proteins [21,22].

The virology of HPV, with a focus on the E6 and E7 genes and the mechanisms of DNA integration, provides critical insights into the pathogenesis of HPV-associated cancers. The dysregulation of cellular processes by viral oncoproteins and the integration of HPV DNA into the host genome contribute to the malignant transformation of infected cells. Ongoing research aimed at unraveling the intricacies of HPV biology holds promise for the development of innovative preventive and therapeutic strategies to mitigate the impact of HPV-associated diseases on global public health.

### 1.2. The Role of HPV in Skin Cancers

Human papillomavirus (HPV) has long been recognized as a major player in the development of various cancers, particularly cervical cancers. However, recent research has shed light on the involvement of certain HPV types in skin tumors, adding a new dimension to our understanding of the virus’s pathogenic potential.

While the HPV types predominantly associated with skin tumors differ from those linked to cervical cancer [23], such as HPV-5, HPV-8, and HPV-15, it is essential to note that HPV infection is not the sole causative factor for skin tumors [24]. Studies have highlighted the presence of HPV in skin tumors, particularly in squamous-cell carcinomas, suggesting a possible correlation between HPV infection and the pathogenesis of these cutaneous neoplasms [4,5,25].

The precise role of HPV in the pathogenesis of skin tumors is still a subject of extensive research. However, several potential mechanisms have been proposed. HPV may integrate into the host genome, disrupting cellular regulation and contributing to neoplasm transformation. Moreover, certain HPV variants can produce oncogenic proteins that interfere with cellular control mechanisms, fostering uncontrolled proliferation of skin cells [26].

Cutaneous squamous-cell carcinomas (SCC) are among the most common skin tumors, and studies have detected the presence of HPV in a significant percentage of cases. The association between HPV and SCC suggests that HPV infection may contribute to the development of some skin tumors. 

HPV virus has also been recognized in the development of anal and oropharyngeal cancers; therefore, HPV infection of the anal and oropharyngeal sites should not be neglected, especially in people at high risk of infection, such as patients at sexually transmissible infection clinics [27].

In a recent study by Baek et al., the authors detected a higher frequence of HPV in pelvic region BDs than in non-pelvic areas, suggesting that sexually transmitted mucosal α-HPV plays a significant role in the pathogenesis of BD, especially in the pelvic region [28].

The presence of HPV in SCC samples has been identified through advanced diagnostic techniques, including viral DNA amplification, sequencing, and immunohistochemical analysis.

While the involvement of HPV in BCC is less apparent compared to SCC, some research has reported the presence of the virus in a fraction of basal-cell tumors [29]. This discovery has prompted further studies to better understand the specific involvement of HPV in different types of skin tumors and the mechanisms through which the virus might contribute to their pathogenesis.

The possible role of HPV has also been investigated in other tumors, such as melanoma. Up to now, there are no data associating oncoviruses with cutaneous melanoma (CM), but in a recent retrospective study, the presence of HPV and Epstein–Barr virus (EBV) was linked with mucosal and ocular melanomas. Although the available data do not support a primary role of oncoviruses in melanoma carcinogenesis, the finding of HPV and EBV DNA in a considerable fraction of mucosal and ocular melanomas suggests that these viruses may act as cofactors in the development of extra-CMs [30].

The presence of HPV in skin tumors has significant implications for diagnosis and treatment. Confirming an HPV infection in samples of cutaneous tumors can influence diagnostic and therapeutic decisions, leading to a more personalized and targeted approach. Antiviral treatments may be explored as therapeutic options in addition to traditional modalities to enhance the effectiveness of therapeutic protocols [31].

Understanding the role of HPV in skin tumors is a rapidly evolving field. Further research is needed to precisely delineate specific pathogenetic mechanisms, the HPV variants involved, and the clinical implications of this association. Larger and more in-depth epidemiological studies could contribute to establishing a clearer link between HPV infection and skin tumors, opening new opportunities for the prevention and management of these neoplasms.

### 1.3. Immunosuppression and HPV: A Common Factor for Keratinocyte Skin Cancer Development?

HPV has emerged as a significant concern in immunocompromised individuals, particularly in the context of immunosuppression, where the risk of developing various cancers, including skin tumors, is notably increased. 

Immunosuppression, whether due to medical interventions such as organ transplantation, human immunodeficiency virus (HIV) infection, or other immunocompromising conditions, is widely recognized as a significant risk factor for the development of HPV-related malignancies [32,33]. The immune system plays a pivotal role in controlling and eliminating HPV infections. When this defense mechanism is compromised, as in immunosuppressed individuals, the risk of persistent HPV infection and subsequent tumor development, including skin tumors, becomes markedly elevated.

Several studies have highlighted a higher prevalence of HPV infections in immunocompromised populations. This increased prevalence is particularly evident in conditions such as solid organ transplantation [34,35], where recipients are often subjected to long-term immunosuppressive therapies to prevent graft rejection. HIV-positive individuals also exhibit higher rates of persistent HPV infections, contributing to a higher incidence of HPV-associated tumors, including those affecting the skin.

The link between HPV and skin tumors is further accentuated in immunocompromised patients. Cutaneous manifestations of HPV-related diseases, such as warts and lesions, are more common and tend to be more persistent in individuals with compromised immune systems. Moreover, the risk of developing more aggressive forms of skin tumors, including squamous-cell carcinomas (SCC), is substantially increased in this population [36].

The management of HPV-related skin tumors in immunocompromised patients involves a multidisciplinary approach. Therapeutic strategies may include antiviral medications targeting HPV, such as topical treatments for cutaneous warts. In cases of malignancy, traditional treatments like surgery, radiation therapy, and topical chemotherapeutic agents may be employed, although the response to these treatments can be variable in immunocompromised individuals.

The intersection between HPV and immunosuppression underscores the importance of understanding the unique challenges faced by individuals with compromised immune systems. As immunosuppression becomes a recognized risk factor for the development of HPV-related skin tumors, clinicians and researchers alike must focus on tailored preventive strategies, early detection measures, and innovative therapeutic approaches to mitigate the impact of these conditions on immunocompromised patients. A comprehensive understanding of the interplay between HPV and compromised immunity is crucial for advancing clinical care and improving outcomes in this vulnerable population.

### 1.4. Prognostic Role, Emerging Therapies, and Future Perspective on HPV

Human papillomavirus (HPV) research has witnessed remarkable progress in recent years, spanning diverse domains from prognostic roles in specific cancers to the exploration of novel therapeutic avenues and associations with a spectrum of diseases. 

Recent studies have significantly advanced our understanding of the prognostic implications of HPV in various cancers [37]. Notably, HPV-positive cervical carcinomas have demonstrated a more favorable prognosis compared to their non-HPV-associated counterparts [38]. The elucidation of the molecular mechanisms underlying this phenomenon is an ongoing area of investigation. Researchers are actively identifying specific biomarkers that could serve as prognostic indicators, offering valuable insights into patient outcomes and informing tailored treatment strategies.

The therapeutic landscape for HPV is evolving rapidly, with a focus on precision and targeted interventions. Inhibitors targeting the DNA topoisomerase enzyme have emerged as promising candidates for the regression of HPV-positive tumors [39]. Gene therapy, particularly utilizing CRISPR-Cas9 technologies, presents a revolutionary approach for tackling persistent HPV infections. By precisely editing viral DNA within host cells, this therapeutic avenue holds great potential for personalized and effective treatment strategies. Additionally, ongoing clinical trials are exploring the efficacy of immunotherapies in enhancing the immune system’s ability to recognize and eliminate HPV-infected cells.

Beyond its well-established role in tumorigenesis, HPV is increasingly being recognized for its potential involvement in other disease processes. Recent investigations have unveiled associations between HPV and certain autoimmune diseases, suggesting a complex interplay between the virus and the immune system [40]. Understanding these connections may pave the way for novel therapeutic interventions targeting both HPV-related conditions and associated autoimmune manifestations. This expanding perspective underscores the need for interdisciplinary collaboration to unravel the intricate links between HPV and various diseases.

The future of HPV research holds tremendous promise, driven by technological advancements and a deeper understanding of the virus’s biology. Genomic approaches have provided unprecedented insights into the molecular intricacies of HPV infections, aiding the identification of novel therapeutic targets. Personalized medicine, fueled by genomic information, is bound to revolutionize the management of HPV-related conditions by enabling tailored treatment regimens based on individual genetic profiles. Additionally, ongoing efforts to enhance vaccination strategies and expand their coverage represent crucial steps in preventing HPV infections and associated diseases.

### 1.5. Is There a Role of HPV in Bowen’s Disease and In Situ SCC?

HPV has long been recognized as a significant player in the etiology of various cancers, particularly those affecting the genital and mucosal regions. Recent research has brought attention to the potential involvement of HPV in the genesis of Bowen’s disease and in situ squamous-cell carcinoma (iSCC) [6,41], both characterized by the abnormal proliferation of squamous cells. According to some new clinical classification, BD and in situ SCC can be differentiated considering their localization and the surrounding skin: the clinical manifestation of BD is an erythematous patch localized on non-chronically photodamaged skin (i.e., arms, forearms, limbs, chest) and not surrounded by other signs of photodamage; differently, in situ SCC is more frequently detected on chronically sun-damaged areas (i.e., face, dorsum of the hand) and surrounded by actinic keratosis or other signs of photodamage. Dermoscopically, they share common criteria, namely glomerular vessels on a reddish background, proving that clinical context is essential to differentiate a form of skin cancer without any risk of progression (BD) from a subtype of SCC with a low but existing risk of progression [8].

Understanding the link between HPV and these tumoral forms is crucial for advancing diagnostic and therapeutic strategies.

Bowen’s disease is a non-invasive form of skin cancer characterized by the presence of atypical squamous cells in the epidermis; in situ SCC represents an early stage of invasive squamous-cell carcinoma, where abnormal cells are confined to the epidermis without infiltrating the underlying tissues. 

The role of UV in the genesis of SCC is crucial; UV radiation can damage DNA directly or through reactive oxygen species (ROS). As a result of UV-driven damage, mitochondrial (intrinsic) and death receptor-mediated (extrinsic) apoptosis mechanisms are activated in keratinocytes, particularly by mitogen-activated protein kinases (MAPK, JNK, and p38) and the tumor suppressor protein p53 [42].

While exposure to ultraviolet (UV) radiation has been a well-established risk factor for these skin conditions, recent studies have sparked interest in the potential role of HPV in their pathogenesis.

Several HPV genotypes, especially those belonging to the high-risk category, have been identified in skin lesions, including Bowen’s disease and in situ SCC. The virus’s DNA has been detected within affected cells, suggesting a potential role in the initiation or progression of these precancerous lesions. Notably, the mechanisms by which HPV contributes to the development of Bowen’s disease and in situ SCC are not fully elucidated, necessitating further investigation.

The proposed link between HPV and skin cancer raises questions about the interplay between viral factors and host cellular processes. It is hypothesized that HPV may interfere with the normal regulation of cell growth and differentiation, leading to the dysregulation observed in Bowen’s disease and in situ SCC. Additionally, the virus’s ability to evade immune surveillance may contribute to the persistence of infected cells and the progression to more advanced stages of skin cancer.

To shed light on these intriguing associations, this study was conducted, aiming to investigate the prevalence of specific HPV genotypes in patients diagnosed with Bowen’s disease and in situ SCC, comparing the results with some controls. This study employed advanced molecular techniques to detect and characterize HPV DNA within skin lesions. 

## 2. Patients and Methods

This study includes 24 cases of in situ SSC and 23 of Bowen’s disease, previously used in published studies for the detection and analysis of HPV alpha in the lesions [43,44]. Controls included in the present study were related to punch biopsies of non-neoplastic inflammatory skin lesions, including dermatitis (17), lupus (2), pseudo-lymphoma (6), lichenoid lesions (6), and eczema (3). Briefly, DNA was extracted from formalin-fixed and paraffin-embedded biopsies using the QIAamp DNA FFPE Tissue Kit following the manufacturer’s instructions. After isolation, DNA was quantified by NanoDrop 1000 (Thermo-Fisher Scientific, Waltham, MA, USA) and submitted to quality assessment by the use of the PCR multiplex specimen control size (IdentiClone™, Invivoscribe Inc., San Diego, CA, USA), which coamplifies four human housekeeping genes of different lengths (100, 200, 300, 400 bases). PCR products were analyzed by capillary electrophoresis. The presence of Beta and Gamma HPV DNA was investigated by PCR using 200–400 ng of DNA and the FFPE1-Fw/FFPE3-Rv consensus primers [45], which are expected to amplify a 158 bp fragment of the E1 ORF in a wide spectrum of HPV types from both HPV genera. In our assays, the FFPE1-Fw and FFPE3-Rv primers were 5′ tagged with the Universal Multiplicom tail (UMT) A and UMT B, respectively [46], generating the primers UMT-FFPE1-Fw 5′-AAGACTCGGCAGCATCTCCATWIYWGHIYTAAAACGAAAGT-3′ and UMT-FFPE3-Rv, 5′-GCGATCGTCACTGTTCTCCASAWWAGWATYTKCAGYTTCAT-3′, with the underlined sequences representing the Universal Multiplicom tail and the plain-character sequences representing the FFPE primers. 

Modified primers yielded more robust amplifications and reliable sequencing results than unmodified FFPE1-Fw/FFPE3-Rv ones. Amplification products were run on 2% agarose gel and visualized by ethidium bromide staining. Bands of the expected size were excised from the gel, and DNA was extracted using the QIAquick Gel Extraction Kit. One microliter of the eluted DNA was used for a second round of amplification with the UMT-FFPE primers (30 cycles) and the PCR product used for standard Sanger sequencing at the sequencing facility (Eurofins Genomics srl, Milan, Italy). Sequences were assessed using the online BLAST^R^, blastn suite (https://blast.ncbi.nlm.nih.gov, accessed on 23 October 2023).

### Statistical Analysis

Associations between clinical data, demographic data, and HPV categories were tested for significance using the chi-square test (or Fisher’s exact test, depending on the size of the groups) for categorical variables. For continuous variables, parametric Student’s t-test was used. All *p* values are two-sided with values ≤ 0.05 regarded as statistically significant. Statistical analyses were performed with the Stata/SE 16 package (Stata, College Station, TX, USA).

## 3. Results

### 3.1. Demographical Data

**Figure 1:**
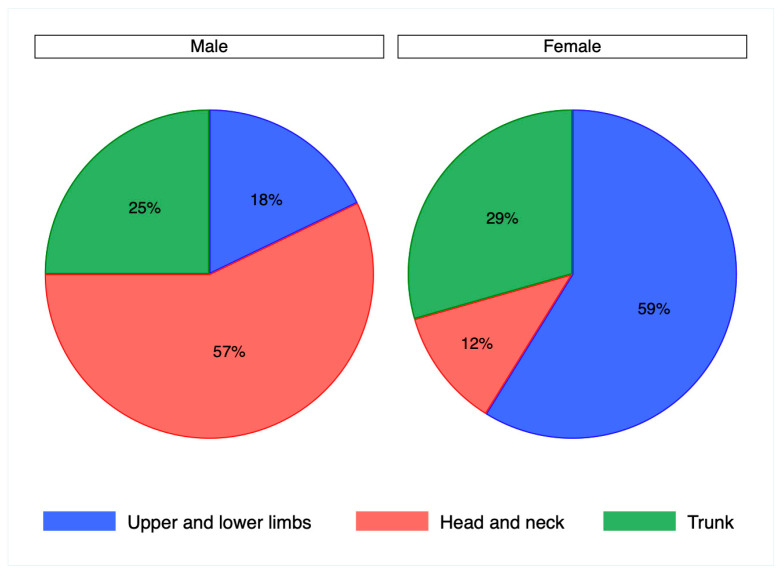
Pie chart representing the distribution of anatomical sites by gender in patient cases, chi squared test (*p* = 0.004).

The cohort of samples analyzed in the present study comprised 23 cases of Bowen’s disease, 24 cases of in situ SCC, and 34 controls. The distribution of genders between cases and controls was similar (*p* = 0.7), with controls including 20 men and 14 women and cases 29 men and 18 women. The mean age of cases was 79 years, with a range of 52–94, without significant differences between genders (*p* = 0.2). The mean age of controls was 61 years (range 32–88), with no differences between genders (*p* = 0.1). The anatomical sites where the lesions occurred in cases and controls were grouped into the head and neck, lower and upper limbs, and the trunk, with the exclusion of anal and perianal regions from this study. According to this subdivision, the distribution of anatomical sites did not vary significantly between cases and controls (*p* = 0.08) as reported in Table 1, but was significantly different between genders (*p* = 0.01). This observation was confirmed in cases (*p* = 0.004) with a higher frequency of lesions on the head and neck in men and on the lower and upper limbs in women, as reported in Figure 1, but not in controls (*p* = 0.8).

### 3.2. HPV

No significant differences were obtained in the distribution of HPV positivity in cases and controls. In detail, HPV detection by PCR was positive in 19 cases (40%) and 14 controls (41%) (*p* = 0.9). HPV positivity did not differ according to gender, both in cases, with eleven men (38%) and eight (44%) women (*p* = 0.7) positive for HPV, and in controls, with eight men (40%) and six women (43%) (*p* = 0.9). Genotyping of HPV was successful in 15 cases, where exclusively the gamma genera were detected, and in 10 controls, including 5 beta HPV and 5 gamma HPV (*p* = 0.01). HPV genotypes in the overall cohort did not differ with respect to the patients’ gender (*p* = 0.7) and anatomical site (*p* = 0.6). Based on previous results, high-risk HPV 16 was detected in two in situ SCC cases; of these, one was also positive for gamma HPV, specifically HPV mSK 046 (species gamma UC).

Considering HPV positivity only in cases with successful genotyping, 15 cases (32%) and 10 controls (29%) (*p* = 0.8) turned out positive, with no significant difference in the distribution of HPV positivity between cases and controls (*p* = 0.8). Even by restricting the positivity of HPV to genotyped cases, the distribution of HPV did not differ by gender, both in cases, with nine men (31%) and six women (33%) (*p* = 0.9), and controls, with five men (25%) and five women (36%), (*p* = 0.7).

**Figure 2:**
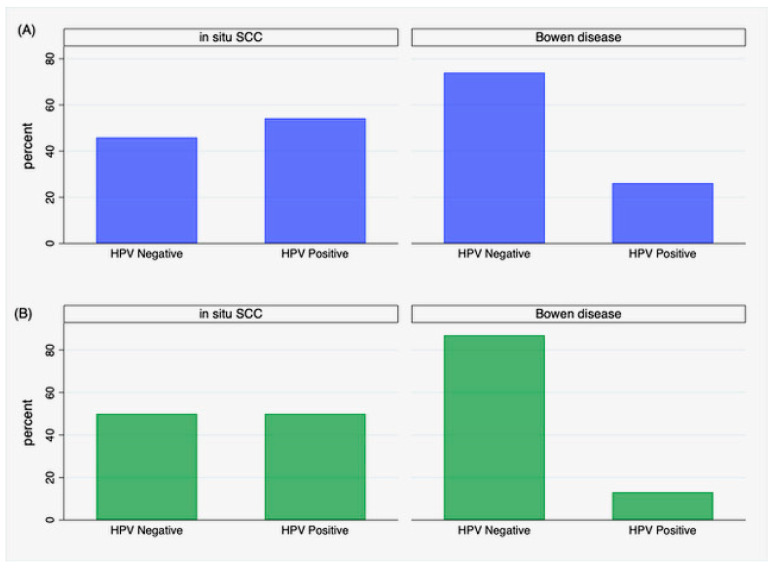
Distribution of HPV positivity in iSCC and Bowen’s disease: (**A**) positivity by PCR detection without genotyping, (**B**) positivity restricted to genotyped cases.

Focusing on cases, a significant difference was found in the distribution of HPV positivity between in situ SCC and Bowen’s disease. HPV was detected in thirteen patients with in situ SCC (54%) and in six (26%) with Bowen’s disease (*p* = 0.05). Restricting results to cases with successful HPV genotyping, this difference was even more evident (*p* = 0.007), including twelve patients (50%) with in situ SCC and three with Bowen’s disease (13%), as depicted in Figure 2B. The distribution of HPV positivity was unrelated to the anatomical site where the lesions occurred (*p* = 0.5), even restricting positivity to genotyped HPV cases (*p* = 0.2).

## 4. Discussion

The results obtained in our study highlight that there are no significant differences in the detection of non-pathological HPV in cases and controls. Gamma and beta HPV have been reported to have high tropism for the skin, supporting in part our data [26]. High-risk HPV was detected only in two cases of in situ SCC [26]. Nevertheless, our data show that BD has a lower rate of HPV detection than in situ SCC. 

In addition to the possible etiologic role, HPV detection in non-melanoma skin cancers might provide the groundwork for the development of alternative treatment measures. For example, HPV vaccination has shown promising results as a treatment measure in patients with cutaneous warts, recurrent respiratory papillomatosis, basal-cell carcinomas, and squamous-cell carcinomas. Wenande et al. successfully administered a nonavalent HPV vaccine to a population of 12 immunocompetent patients with a mean age of 76.2 years affected by actinic keratoses. They concomitantly administered topical therapies for actinic keratoses such as cryotherapy, diclofenac, photodynamic therapy, and 5-fluorouracil. Twelve months after the first dose of the vaccine, an average 85% reduction in total actinic keratosis burden was recorded, highlighting a possible role of HPV vaccine in the reduction of AK recurrence [47].

In addition to its therapeutic role, the presence of HPV in skin cancers may also have a classificatory and diagnostic role. Regarding the first point, many keratinocytic tumors are often referred to by interchangeable names, such as in situ SCC and Bowen’s disease or well-differentiated SCC and keratoacanthoma, without a precise difference. This heterogeneous nomenclature can lead to confusion in the definition of the neoplasm; for this reason, some authors have proposed to distinguish these tumor forms on the basis of the presence or absence of the field of cancerization; for example, well-differentiated SCC appears on photodamaged skin and/or is surrounded by actinic keratoses while keratoacanthoma appears on even, non-photoexposed skin; it appears evident that while in the case of well-differentiated SCC, the main risk factor is UV radiation, for keratoacanthoma, on the other hand, a viral etiopathogenesis has been hypothesized; in fact, the search for HPV DNA showed very high rates with statistically significant differences compared to SCC; with a view, therefore, to demonstrate the same concept for in situ SCC and BD, the study described above was conducted. Contrary to our initial hypothesis, namely that in the case of in situ SCC, UV-mediated etiology would be greater and in BD, there would be a predominant role of HPV, our results disproved our initial hypothesis and even supported a contrary thesis. Our results can find explanation in the well-known immunosuppressive activity of UV radiation [48].

Consequently, due to UVR immunosuppression, in situ SCC in photoexposed sites could be more prone to infectious agents, such as HPV, than BD in photoprotected areas. Although gamma and beta HPV have not been associated with any risk of the development of skin tumors, they could be relevant to therapy decisions and/or prognosis. Our conflicting results allow us, indeed, to explore a secondary role of HPV in skin tumors, which was previously mentioned, namely its prognostic role. In other forms of neoplasms, such as oropharyngeal tumors, the presence of HPV has become part of the neoplasm classification system because a positive prognostic role has been demonstrated in cases of related HPV neoplasms. In fact, the high presence of HPV in KA and in situ SCC correlates with forms of neoplasms, namely with small keratinocyte invasion, resolvable with easy surgical interventions or topical/intralesional therapies. For this reason, although HPV cannot be assigned an etiopathogenetic role, further studies on even the most advanced forms will be necessary to assess whether it is a positive prognostic factor, especially in relation to the therapeutic response to immunotherapy, to which invasive forms of SCC show a very high response rate.

Anyway, we acknowledge that our study has some limitations. The main limitation is the small sample size, which does not allow us to prove the negation of our first hypothesis, even if it represents a starting point for future research to confirm the results of our pilot study. Another point to consider is the heterogeneity in the naming of non-melanoma skin cancer; according to WHO classification, BD and iSCC can be used as synonyms, but according to some proposals, the term BD should be used for HPV-induced tumors of the genital area. Differently, for other research groups, BD is a term used to refer to tumors that arise in the absence of the field of cancerization. From a histological point of view, BD/SCC can be used as synonyms; that said, a limitation of our study consists of having only one pathologist evaluate the samples histologically. In future studies, at least two pathologists should be enrolled for a consensus diagnosis and naming of samples. 

## 5. Conclusions

Contrary to previous hypotheses regarding the role of HPV as an etiopathogenetic cofactor of BD, a higher percentage of the virus was found in iSCC. Genital BD, where the role of HPV is well known, were excluded from this study, and no significant difference in the expression of HPV was observed based on extragenital anatomical localization. To date, few cases of extragenital BD positive for HPV have been described, mostly in acral areas such as the hand and foot or associated with immunosuppressive treatments [49,50]. Specific iSCC data are missing because of the lack of an equal taxonomy in all studies performed. Often, in fact, the terms BD and iSCC are used as synonyms without distinguishing the area of occurrence and the association with a field of cancerization. Our study, which aimed to highlight the predominant role of UV rays in the etiopathogenesis of iSCC and HPV in BD, did not prove its initial hypothesis, suggesting neither an etiopathogenetic nor a cofactor role of the virus in these tumors. A possible explanation can be found in the biological behavior of these neoplasms: the only lesion that grows exponentially is KA, in whose case the literature data agree that there is a high detection rate of HPV; in the other tumor forms (SCC, iSCC, and BD), the rate of HPV detection is lower. According to these results, HPV probably plays a role only in the rapid and exponential growth of keratinocyte neoplasms; however, further studies on the subtypes of fast-growing SCC are needed to confirm this hypothesis, especially in locally advanced forms for which no specific data are available to date. This hypothesis, if confirmed, could explain the rapid growth of keratinocyte skin tumors and could be used as a screening test on histological samples to identify fast-growing and aggressive forms.

## Figures and Tables

**Table 1:** Anatomical sites of cases and controls.

Anatomical Sites	Cases (%)	Controls (%)
Upper and lower limbs	15 (32%)	18 (53%)
Head and neck	18 (38%)	6 (18%)
Trunk	12 (26%)	10 (29%)
Not available	2 (4%)	0 (0%)
Total	47 (100%)	34 (100%)

## Data Availability

Data supporting this study are made available by authors on request.
